# Prevalence of scars: an international epidemiological survey in adults

**DOI:** 10.1111/jdv.18277

**Published:** 2022-06-07

**Authors:** J.M. Amici, C. Taïeb, C. LeFloc'h, A.‐L. Demessant‐Flavigny, S. Seité, O. Cogrel

**Affiliations:** ^1^ Dermatology Department, CHU Bordeaux Hôpital Saint‐André Bordeaux France; ^2^ Patients Priority European Market Maintenance Assessment Fontenay‐sous‐Bois France; ^3^ La Roche‐Posay Levallois‐Perret France; ^4^ Groupe Chirurgical de Dermatologie French Society of Dermatological Surgery Paris France

*Dear Editor*,

Skin is the most vulnerable organ that is constantly exposed to potential injury, and wound healing is a vital process for the survival of all higher organisms.^1^ Scarring is most noticeable in the skin, but it also affects almost all adult mammalian and human tissues and organs.^2^ They may result from surgery, skin injury, burning or inflammatory skin diseases, such as acne, chickenpox or shingles.^3^ Scars may be extensive, dystrophic, appearing on areas not covered by clothes and considered embarrassing. They may also be associated with symptoms such as pruritus, pain or other discomforts. According to a study conducted in the United Kingdom, 26% and 44% of patients reported pain and itching related to their scars, impacting their physical comfort and functioning.^4^ Usually, scars are composed of loose fibrous connective tissue and are remodelled during healing.^5^ Chronic inflammation of the dermis and uncontrolled activation of myofibroblasts may result in abnormal scar overgrowth leading to a hypertrophic scar or a keloid with an excess of extracellular matrix proteins.^6^ Despite being common throughout the world, the epidemiology of scars has not yet been properly investigated.

The aim of this international epidemiological study was to assess the prevalence of scars worldwide. The present article describes the general population with participants reporting at least one scar having appeared during the year prior to this study.

Our participants were selected *via* a stratified random sampling method of internet users who agreed to participate in panel surveys. Data about sociodemography, presence, origin and symptoms of scars using an internet survey were collected between April and May 2020. The survey focused on the most recent scars reported in order to simplify data collection.

Overall, 11 100 individuals from Brazil (2000), China (3050), France (2000), Russia (2000) and the United States (2050) answered the survey; 48.5% of subjects reported at least one scar, and in 22%, the most recent scar was less than one‐year‐old. The most often reported location of recent scars in women was the abdomen (20.4%) and face (15.9%); in men, it was the face (18.7%) and abdomen (13.2%). Significantly more men (13.3%, *P* < 0.00001) than women (8.4%) reported scars on their hands. For 50.8%, the origin of the most recent scar was accidental or due to a disease (women: 50.8%, men: 60.5%, *P* < 0.0001); 35% of women and 28% of men indicated that general or orthopaedic surgery caused the scarring (*P* < 0.0001). Details are given in Table 1. Overall, 12.3% of men and 10.7% of women who reported scars also reported pain (*P* < 0.03). Table 2 provides details about symptoms. In 2014, international guidelines on the management of scars were issued. However, these guidelines have not yet been updated.^7^, ^8^ Currently, some procedures are available to manage scars, such as intralesional injections of corticosteroids and/or 5‐fluorouracil, cryotherapy, radiotherapy, laser therapy and surgical interventions, together with additional measures such as sun protection, silicone‐based dressings or gels.^9^, ^10^ Scar management mainly consisted in the use of topical products such as healing creams (13.2%), antiseptic solutions (11.2%) or a topical antibiotic (11.9%). Medical care was significantly (*P* < 0.001) more frequently provided to subjects with painful scars.

**Table 1:** Sociodemographic data and general data about scars

	Global		Men		Women	
	N = 11 100		N = 5486		N = 5614	
Age	43.0 ± 14.9		42.8 ± 14.9		43.2 ± 14.9	
mean ± SD						
	n	%	n	%	n	%
**Age range (years)**						
18–24	1367	12.32	694	12.65	673	13.67
25–34	2430	21.89	1214	22.13	1216	24.30
35–44	2289	20.62	1126	20.52	1163	22.89
45–54	2093	18.86	1036	18.88	1057	20.93
55–64	1781	16.05	893	16.27	888	17.81
>65	1140	10.27	523	9.53	617	11.40
**Mean number of scars ± SD**	4.1 ± 4.9			4.0 ± 4.5		4.2 ± 5.0
**Number of scars**						
< one year	1196	22.2	602	22.7	594	21.7
> one year	4186	77.8	2046	77.3	2140	78.3
**Time since presence, if > one year (years)**	12.4 ± 12.7		12.5 ± 13.0		12.3 ± 12.4	
**Origin of scars**						
Accident	2992	55.59	1604	60.57	1388	50.77
General/orthopaedic surgery	1712	31.81	741	27.98	971	35.52
Skin excision	299	5.56	132	4.98	167	6.11
Restorative/cosmetic surgery	251	4.66	125	4.72	126	4.61
Cosmetic procedure	128	2.38	46	1.74	82	3.00
**Type of scars**						
Hyperthropic or keloid scar	671	12.47	335	12.65	336	12.29
Did not know	1897	35.25	1897	35.25	949	34.71

SD, standard deviation.

**Table 2:** Prevalence of clinical symptoms

	Global			Men			Women		
	n	%	Intensity>5 (0–10)	n	%	Intensity>5 (0–10)	n	%	Intensity>5 (0–10)
**Presence of cracks or fissures**	1774	32.96%	10.80%	963	36.37%	12.61%	1198	43.82%	10.80%
**Visible detachment of thin skin flakes**	2067	38.41%	15.09%	1094	41.31%	17.03%	736	26.92%	15.09%
**Redness**	2712	50.39%	20.09%	1389	52.45%	21.30%	1192	43.60%	20.09%
**Drought or dryness**	2519	46.80%	19.18%	1279	48.30%	20.43%	1240	45.35%	19.18%
**Pruritus**	2446	45.45%	17.58%	1254	47.36%	18.73%	1323	48.39%	17.58%
**Burning sensation**	1631	3030%	10.18%	895	33.80%	12.01%	973	35.59%	10.18%
**Pulling**	2453	45.58%	18.80%	1255	47.39%	19.86%	811	26.66%	18.80%

To our knowledge, this is the first international epidemiological survey on the prevalence, origin, location and impact of scars. Further investigations need to be performed.

## Funding sources

This project was funded by La Roche Posay.

## Conflict of interest

S. Seité, AL Demessant‐Flavigny and C. LeFloc'h are employees of La Roche‐Posay.

## Data Availability

The data that support the findings of this study are available from the corresponding author upon reasonable request.
